# Spatial Intelligence and Multi‐Omics in Alzheimer's Disease Therapeutics. The Transformative Potential of the 3DNasal Project.

**DOI:** 10.1002/alz70859_099006

**Published:** 2025-12-25

**Authors:** Bisschops G Bisschops

**Affiliations:** ^1^ Neuroteg AI, Kasterlee, Antwerp Belgium

## Abstract

**Background:**

Alzheimer's Disease (AD) is influenced by an intricate interplay of genetic, metabolic, and microbial factors. Recent studies highlight the critical roles of the gut‐brain axis, lipid metabolism, bile acids, and systemic inflammation in AD progression. The 3DNasal project integrates spatial intelligence, biofilm technology, and multi‐omics insights to develop a scalable, precision‐based therapeutic platform targeting AD’s multifactorial pathology.

**Method:**

Objective: To design a biofilm‐based nasal spray incorporating findings from metabolomics, microbiome research, and genomics (e.g., Dilmore et al., 2024; Mohanty et al., 2024) to restore microbiome homeostasis, reduce neuroinflammation, and enhance systemic resilience against AD progression.

**Result:**

Preclinical findings demonstrate improved biofilm adhesion, enhanced delivery to the olfactory bulb, and significant modulation of the gut‐brain axis. Data reveal that the Mediterranean Ketogenic Diet mitigates AD risk factors through serum and CSF metabolomics (Schweickart et al., 2024). Multi‐omics analyses further identify novel microbial and metabolic signatures associated with AD pathology (Dilmore et al., 2024) and systemic inflammation (Liang et al., 2024)

**Conclusion:**

The 3DNasal project represents a novel therapeutic paradigm by integrating spatial intelligence, multi‐omics, and bioengineering. This precision‐based approach targets localized and systemic drivers of AD, paving the way for scalable and adaptive interventions. Future research will focus on clinical validation, adaptive personalization, and exploration of lifestyle‐microbiome interactions to optimize therapeutic outcomes.

